# Evaluation of right heart function in a rat model using modified echocardiographic views

**DOI:** 10.1371/journal.pone.0187345

**Published:** 2017-10-31

**Authors:** Ivan Bernardo, James Wong, Mary E. Wlodek, Ross Vlahos, Paul Soeding

**Affiliations:** 1 School of Health and Biomedical Sciences, RMIT University, Bundoora, Australia; 2 Department of Cardiology, Royal Melbourne Hospital, Parkville, Australia; 3 Department of Physiology, University of Melbourne, Parkville, Australia; 4 Cardiovascular Therapeutics Unit, Department of Pharmacology, University of Melbourne, Parkville, Australia; 5 Department of Anaesthesia and Pain Management, Royal Melbourne Hospital, Grattan St, Parkville, Australia; Worcester Polytechnic Institute, UNITED STATES

## Abstract

Echocardiography plays a major role in assessing cardiac function in animal models. We investigated use of a modified parasternal mid right-ventricular (MRV) and right ventricle (RV) outflow (RVOT) view, in assessing RV size and function, and the suitability of advanced 2D-strain analysis. 15 WKY rats were examined using transthoracic echocardiography. The left heart was assessed using standard short and long axis views. For the right ventricle a MRV and RVOT view were used to measure RV chamber and free wall area. 2D-strain analysis was applied to both ventricles using off-line analysis. RV chamber volume was determined by injection of 2% agarose gel, and RV free wall dissected and weighed. Echocardiography measurement was correlated with necropsy findings. The RV mid-ventricular dimension (R1) was 0.42±0.07cm and the right ventricular outflow tract dimension (R2) was 0.34±0.06cm, chamber end-diastolic area measurements were 0.38±0.09cm^2^ and 0.29±0.08cm^2^ for MRV and RVOT views respectively. RVOT and MRV chamber area correlated with gel mass. Doppler RV stroke volume was 0.32±0.08ml, cardiac output (CO) 110±27 ml.min^-1^ and RV free wall contractility assessed using 2D-strain analysis was demonstrated. We have shown that modified MRV and RVOT views can provide detailed assessment of the RV in rodents, with 2D-strain analysis of the RV free wall potentially feasible.

## Introduction

Animal models provide valuable insight into the pathophysiology of chronic lung disease, and enable assessment of potential therapeutics for conditions such as chronic obstructive pulmonary disease (COPD) [1–3]. Animal exposure to chronic hypoxia, monocrotaline injury or pulmonary trunk banding, have contributed greatly to understanding the relationship between lung injury and progressive dysfunction of the right ventricle (RV) [4]. In many studies echocardiography plays a major role in assessing RV function and the efficacy of therapeutic intervention. Transthoracic views of the RV include the short-axis (SAX) mid-ventricular and aortic RV outflow views (RVOT), and the long-axis (LAX) apical 4-chamber view. Recent advances in ultrasound technology, including 2D speckle tracking or strain, have seen these modalities now being applied to clinical practice [5]. However the application of 2D-Strain in small animals is emerging, and has been recently reported for left ventricular assessment [6]. A limitation of transthoracic imaging in small animals is the effect of anatomical artifact from the thoracic cage or liver on image quality. Suboptimal imaging limits RV assessment with standard views, and can prevent the application of advanced analysis including 2D-strain imaging.

In an alternative approach, Scherrer-Crosbie et al developed a transoesophageal technique in mice, where the right ventricular wall and chamber were evaluated in the mid-right ventricular SAX view [7]. Significantly, echocardiographic findings were validated with ultrasound flow-measurement and magnetic resonance imaging (MRI). This modified RV approach is relevant to current clinical guidelines, which advocate the use of a RV-focused view over the standard four-chamber view. Optimal RV assessment occurs when imaging allows visualization of most or entire RV free wall [8].

In this study we aim to adapt the Scherrer-Crosbie mid-right ventricular (MRV) view to transthoracic examination in the rat, and compare RV assessment at the mid-ventricular SAX level, with the aortic SAX view of the right ventricular outflow tract (RVOT). The rat was the rodent of choice as it has a bigger heart than the mouse and it would therefore be easier to measure and detect small changes in size, structure and function. The RVOT view is a valuable window for assessing Doppler-derived RV ejection. In addition we plan to investigate whether the modified MRV view is suitable for 2D-strain analysis.

## Methods

Studies were conducted in accordance with National Health and Medical Research Council of Australia guidelines, and approved by the University of Melbourne Animal Ethics Committee (Ethics No. 1212675). All animals were acquired from the Animal Resource Center, Perth, Australia.

### Echocardiographic analysis of ventricular size and function

**Fig 1:**
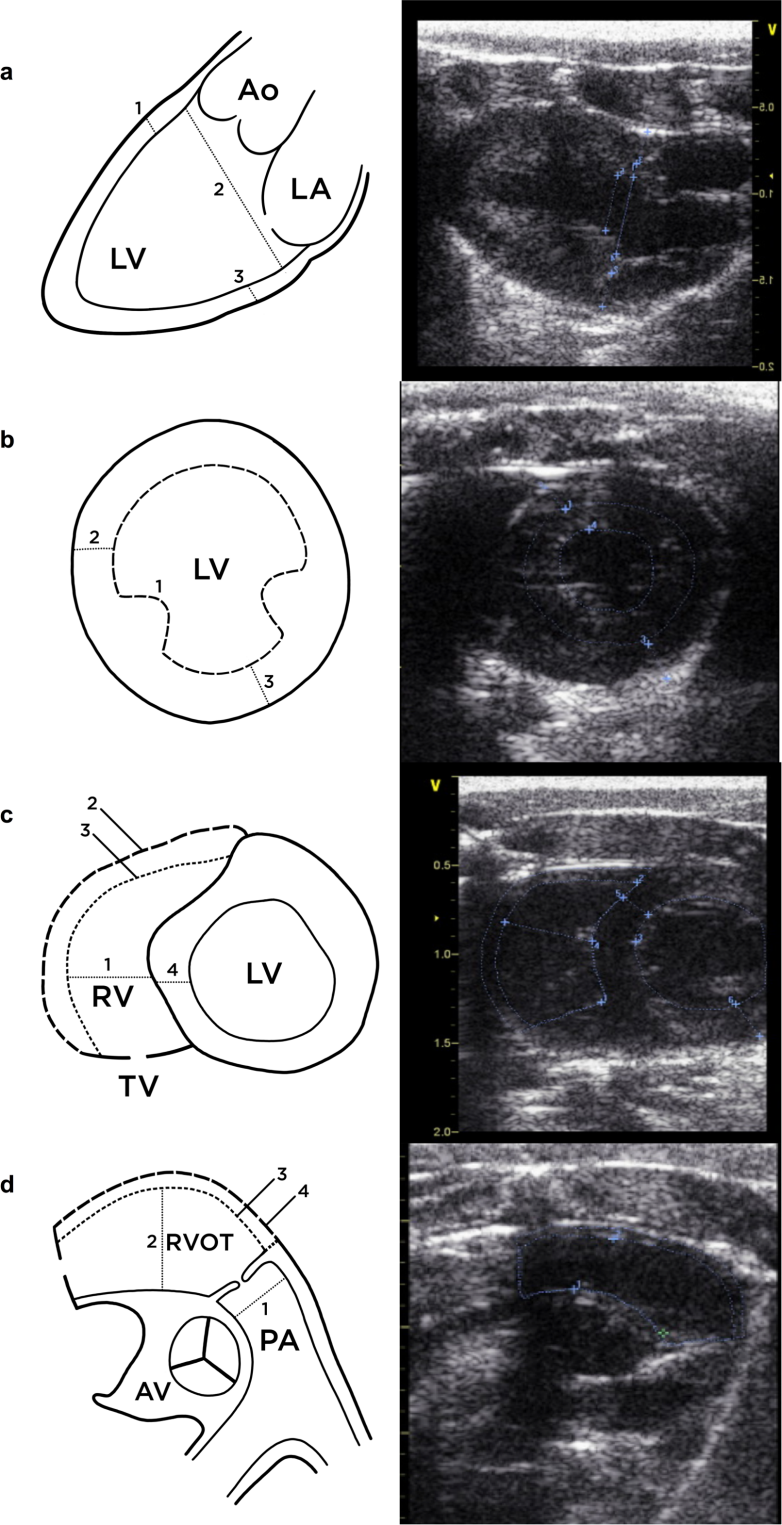
Echocardiographic views used to assess cardiac function. a. Parasternal long-axis (PLAX) view of LV (1. Interventricular septum IVS, 2 End-diastolic dimension LVIDD, 3. Posterior wall dimension PWD). b. Mid-ventricular SAX papillary view (1. LV end-diastolic chamber area LVEDA, 2. IVS, 3. PW). c. Mid-right ventricular (MRV) view, modified from Scherrer-Crosby[7], (1. R1 mid-chamber dimension, 2. Sub-epicardial chamber area RV_epi_, 3. Sub-endocardial chamber area RV_endo_, 4. IVS). d. Short axis (SAX) right ventricular outflow tract (RVOT) view (1. Main pulmonary artery diameter PAΦ, and locus for PWD measurement of ejection velocity time integral VTI 2. RVOT dimension R2, 3. Sub-epicardial RVOT chamber area RVOT_epi_, 4. Sub-endocardial RVOT chamber area RVOT_endo_).

Fifteen male WKY rats (300-350g) were anesthetized with 2.5% isoflurane, spontaneously ventilated and placed on a heated pad in the semi-left lateral position with upright tilt, suitable for echocardiographic examination. Pulse rate, oxygen saturation and temperature were continuously monitored. After shaving, a sequential examination of the left and right ventricles was performed using a Vivid E9 with i13-L (6-14MHz) linear array transducer (GE Vingmed Ultrasound AS, Horten, Norway). Each study imaged the parasternal long axis (PLAX) and mid-papillary SAX views of the left ventricle (LV), and the modified MRV and aortic RVOT/pulmonary artery SAX view of the RV (Fig 1). LV morphology was assessed for interventricular septal (IVS) wall and posterior wall (PW) thickness, LV internal-diastolic dimension (LVIDD), LV internal-systolic dimension (LVISD), fractional shortening (FS), LV end-diastolic chamber area (LVEDA), LV end-systolic chamber area (LVESA) and fractional area change (FAC). RV chamber width was assessed at mid-chamber in the MRV view (R1) and RVOT width measured in the aortic SAX view (R2). In this view the main pulmonary artery diameter (PAϕ) was also measured. RV wall area was measured in both MRV and RVOT SAX views calculated from the difference between traced epicardial and endocardial areas [7]. Right ventricular ejection was measured in the aortic SAX view at the proximal main pulmonary artery (PA diameter) using 2D-Doppler and the waveform analyzed for pulmonary artery velocity-time integral (VTI) and time to peak ejection (PAAT). Pulmonary artery systolic pressure (PASP) was not assessed since analysis of tricuspid regurgitation jet was not performed.

Echocardiographic measurements in this study included:

Fractional Shortening (FS) = LVIDD-LVISD/LVIDD

Fractional Area Change (FAC) = LVEDA-LVESA/LVEDA

LV volume/Stroke volume (SV) = based on geometric truncated ellipsoid model[9]

RV Free Wall Area = Epicardial area–endocardial area

RVOT Free Wall Area = Epicardial area–endocardial area

RV ejection stroke volume RVESV = PA area. VTI. HR, where PA area is the cross-sectional area of the main pulmonary artery, VTI is the area beneath the PA pulsed-wave velocity waveform or velocity-time integral and HR is heart rate.

### Strain analysis

Real-time imaging loops of ventricular motion during the cardiac cycle were recorded for off-line analysis. The RV was imaged in the adapted MRV view and the LV in the mid-ventricular SAX view. High frame rates necessitated manual tracing of epicardial and endocardial walls for 2D-strain analysis (Echopac, version 2011, GE Healthcare). Segmental and global wall motion was then analyzed.

### Injection of agarose gel and tissue collection

**Fig 2:**
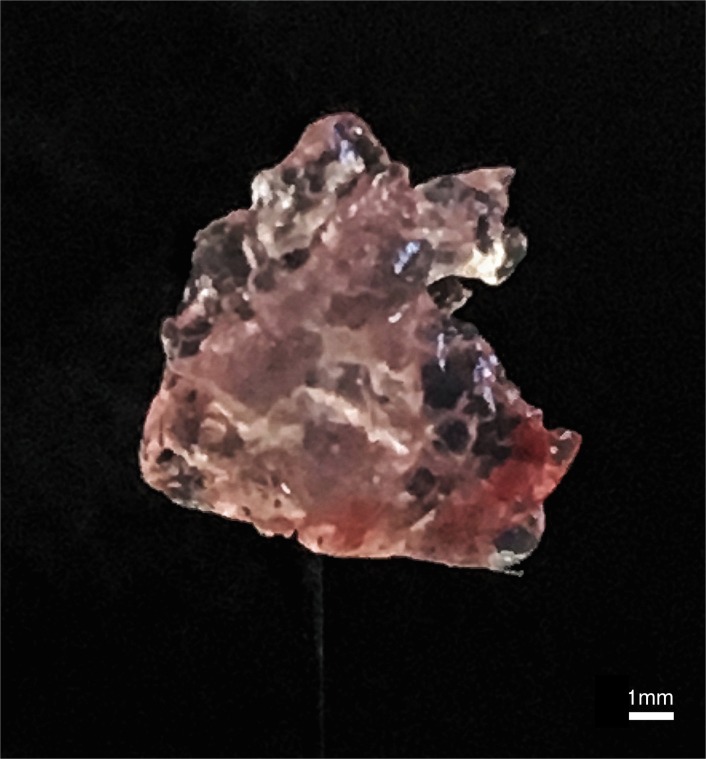
Example of an agarose gel mold of the right ventricle. The molds were extracted following injection and solidification of agarose within the RV chamber.

Animals were euthanized using inspiratory CO_2_, the heart immediately excised and injected with KCl. To correlate RV chamber size and wall thickness with echocardiographic measurement agarose gel (2%, ScientifiX, Australia) was injected (using a 1ml syringe and 21G needle) into the RV chamber (the needle was inserted into the RV where the RV meets the apex of the heart and the gel expelled into the RV until the RV was completed saturated to the point of expansion and gel spill over outside of the heart. This occurred in 100% of animals) and left to fix at room temperature. The gel was suspended in Hank’s Balanced Salt Solution 40 mM HEPES (sHBSS). Following injection (at 10 min) the RV wall was excised from the heart, exposing the hardened agarose gel mould of the RV chamber (Fig 2). The RV free wall was then freely dissected away from the septal wall and atrio-ventricular valves. Both the agarose gel mould and RV free wall were weighed.

### Statistics and analysis

Data are expressed mean ± standard deviation (SD). Linear regression was used for correlation between echocardiographic findings and agarose gel mass, and RV wall mass at necropsy. P<0.05 was taken as significant (Graphpad Prism version 6 was used).

## Results

Echocardiography was performed in 15 animals, and autopsy performed in 14 due to poor image quality in one animal. Animals were of similar age with mean body weight 332 ± 22 g, and heart rate 347 ± 23 bpm. Cardiac measurement of ventricular chamber size and function were within the normal range for our laboratory, and consistent with previous reports [10].

Left ventricle: LVIDD and LVISD were 0.72± 0.09 and 0.37± 0.07cm respectively, and using the hemisphere-cylinder model equation [9], end-diastolic chamber volume was estimated as 0.39 ± 0.08cm^3^. Fractional shortening was 48.25± 8.90%, FAC measured as 72%, and calculated stroke volume 0.20 ± 0.05 ml, indicating normal systolic function.

Right ventricle: due to the geometrical shape of the RV, the MRV view imaged a larger proportion of the RV chamber than the outflow RVOT view. The MRV chamber dimension (R1) was 0.42± 0.07cm, the RVOT dimension (R2) was 0.34± 0.06cm, and chamber end-diastolic area measurements were 0.38± 0.09 cm^2^ and 0.29± 0.08cm^2^ for MRV and RVOT views respectively.

**Fig 3:**
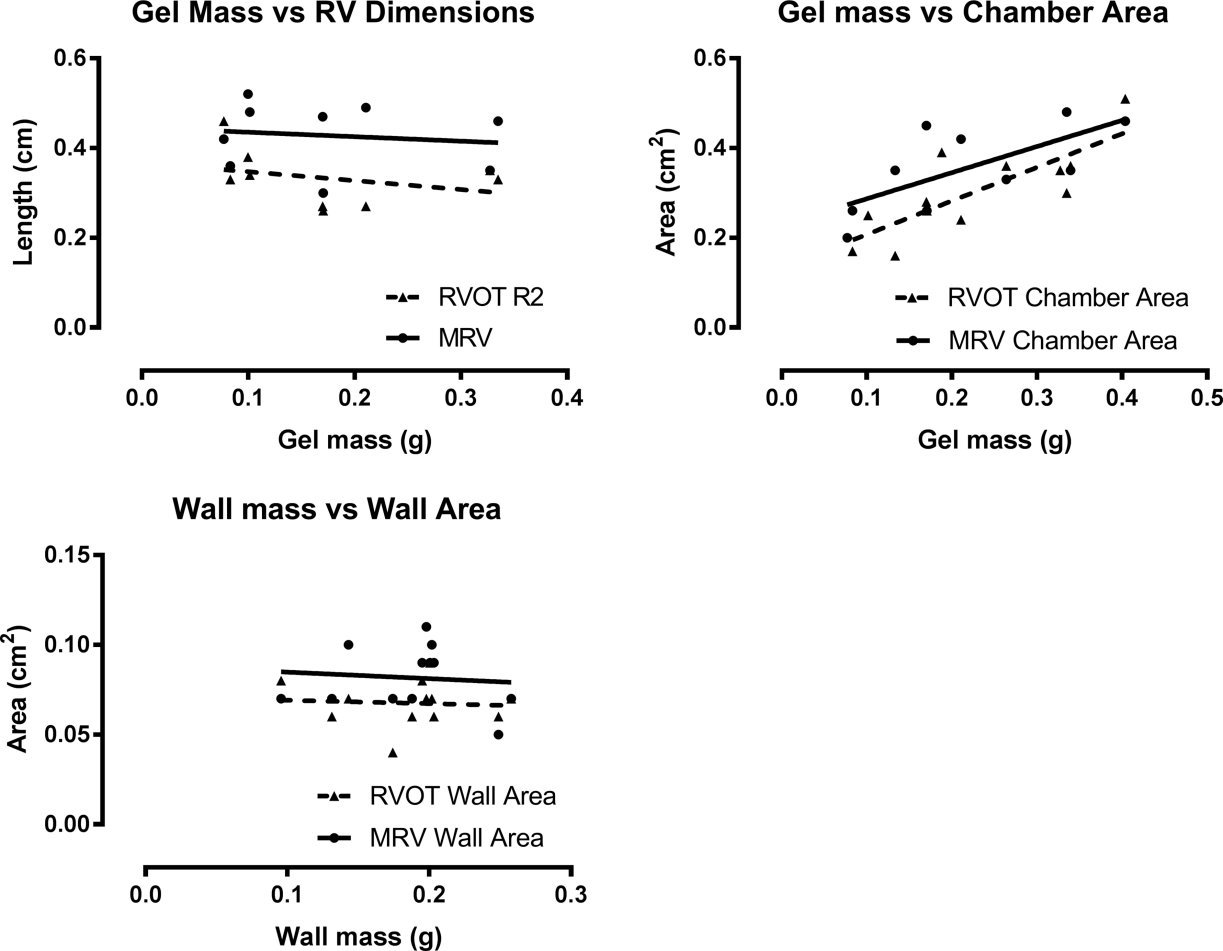
Correlation of RV chamber dimensions with gel mass from necropsy findings. The gel mass was 0.18 ± 0.10g, the RVOT dimension (R2) was 0.34 ± 0.06cm and the mid-ventricular dimension (R1) was 0.42 ± 0.07cm for SC and RVOT views respectively. Neither RVOT nor MRV dimensions correlated with agarose gel mass (R = 0.098, P = 0.41 and R = 0.018, P = 0.73)**.** The chamber area was 0.29 ± 0.08 cm^2^ and 0.38 ± 0.09 cm^2^. Both RVOT and MRV chamber areas correlated with gel mass (R = 0.72, P = 0.02 and R = 0.78, P = 0.03). The RV wall mass was 0.19 ± 0.04g, and the wall area was 0.069 ± 0.017cm^2^ and 0.084 ± 0.017cm^2^ for RVOT and MRV views respectively. Neither RVOT nor SC wall area correlated with RV wall mass (R = 0.004, P = 0.84 and R = 0.009, P = 0.77).

Correlation with necropsy: RV chamber dimension R1 and R2 had no correlation (p = 0.41 and 0.73 respectively), whereas RVOT and MRV chamber area did correlate with gel mass (p = 0.002 and 0.03 respectively, Fig 3). Echocardiographic RV wall area, 0.08±0.02 and 0.07±0.01 cm^2^ for MRV and RVOT views respectively, corresponded to a total RV free wall mass of 0.19±0.04g at necropsy. There was no correlation between wall mass and RV wall area in either view, R 0.004 and 0.009, p = 0.84 and 0.77 respectively (Fig 3).

**Fig 4:**
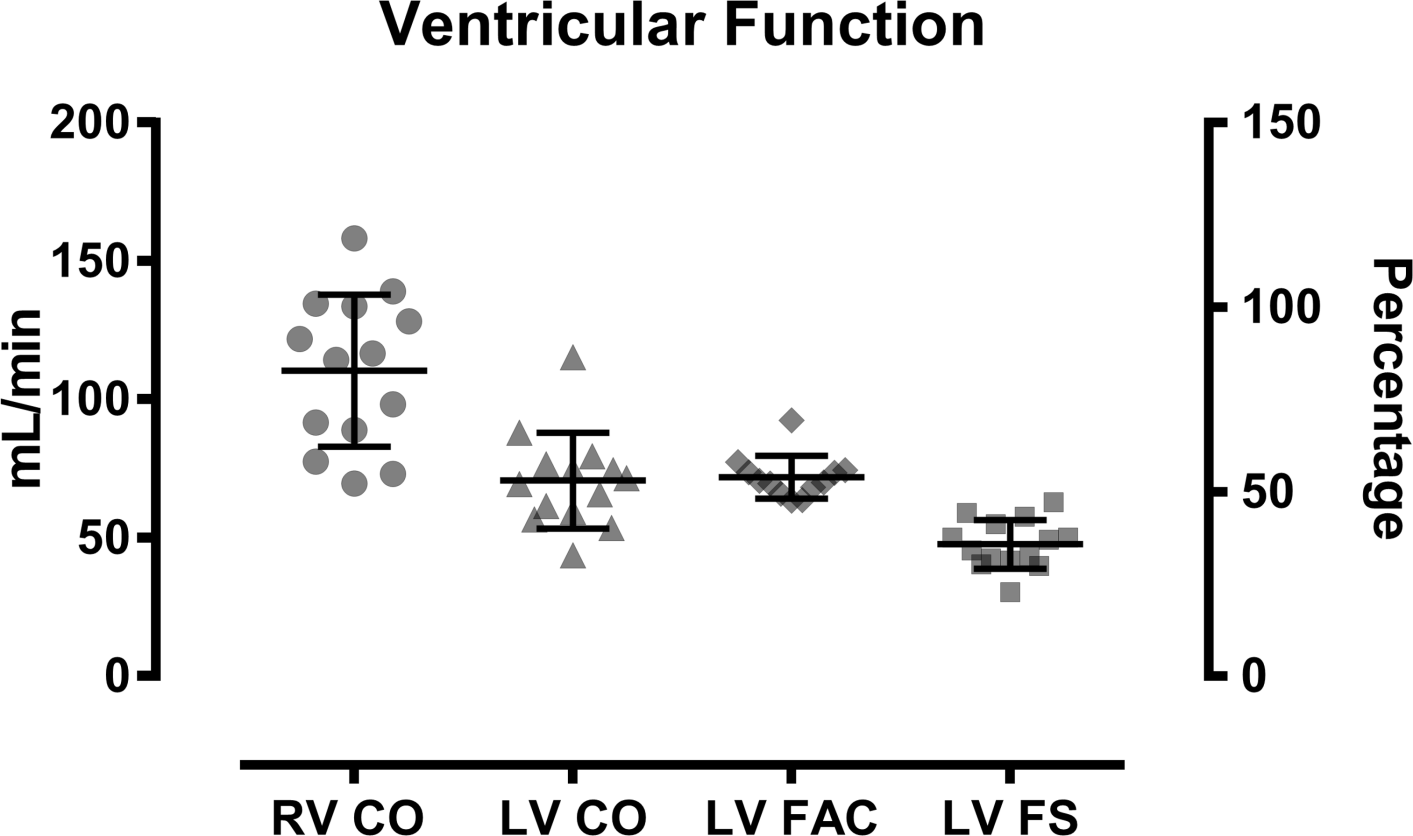
Ventricular function. Ejection indices of cardiac output (RV CO and LV CO), left-ventricular fractional area change (LV FAC) and Fractional shortening (LV FS).

Right Ventricular Ejection. The average RVESV was 0.32±0.08 ml, with estimated RV CO 110 ± 27 ml/min. These values were consistent with cardiac output ranges measured via echocardiography in existing literature [11]. Cardiac output was higher with direct Doppler measurement of RV ejection compared to calculated cardiac output based on LV geometrical change (110 ± 27 ml/min and 70.63 ± 17.29ml/min respectively). Other indices of LV ejection, FAC (72.61 ± 7.92%) and FS (48.25 ± 8.90%), were consistent in measurement (Fig 4).

**Fig 5:**
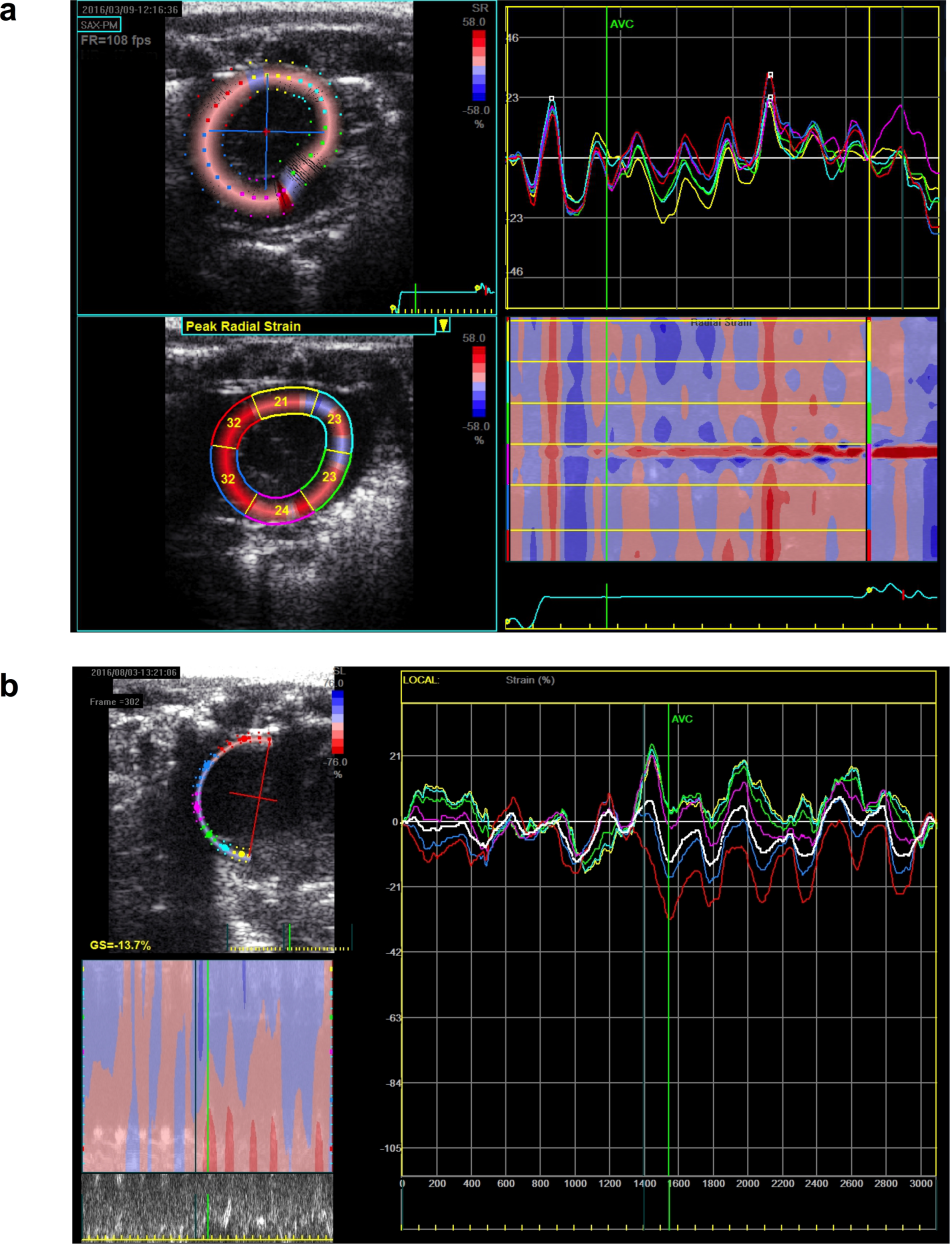
2D-Strain measurement of ventricular contraction. a. Mid-papillary SAX view of left ventricle with segmental wall analysis. b. Similar measurement of right ventricular free wall contraction in MRV view. A rapid HR necessitated manual gating of aortic valve opening and closure (AVC) for segmental deformation measurement during the cardiac cycle.

2D-Strain analysis. Strain analysis was performed in both ventricles. Strain analysis of left ventricular contraction was applied in the SAX view and RV analysis applied in the MRV view (Fig 5). In many rats poor endocardial border definition of the RV limited strain analysis; however, as Fig 5 demonstrates, segmental wall motion analysis was possible in the modified MRV view.

## Discussion

This study has shown echocardiographic measurement of RV chamber area to correlate with RV chamber gel volume in both MRV and RVOT views. Chamber width dimension or RV wall area however, did not correlate with gel volume or wall mass at necroscopy in these normal animals. In pathological states of RV dilatation or hypertrophy, it is possible that correlation with echocardiographic measurement may become evident, as changes in dimension and area are more pronounced. The study has also demonstrated the MRV view to be suitable for strain analysis, since a large area of the RV free wall is easily visualized. Examination with the RVOT view provides further valuable imaging of the RV infundibulum and proximal pulmonary artery, and enables measurement of RV stroke volume using pulsed-wave Doppler. Our data showed stroke volume to be greater (and likely more accurate) with RVOT Doppler measurement, compared to LV measurement based on geometric estimation alone. Further the RVOT view enables analysis of the pulmonary spectral velocity waveform, with measurement of the pulmonary artery acceleration time (PAAT), an index of elevated pulmonary arterial pressure in rodent models [12]. Both views are complimentary, and together, provide a focused but comprehensive assessment of right ventricular structure and function. A focus of this study was to identify a practical approach to RV assessment, particularly when transthoracic imaging is limited in small animals.

Current clinical guidelines advise the use of multiple echocardiographic views, for comprehensive assessment of right ventricular function [8]. This relates to the complex RV anatomy, involving a pyramidal-shaped chamber, with inlet and outlet portions communicating to the right atrium and main pulmonary artery. Unlike the left ventricle, the right ventricular wall comprises myofibrils predominantly oriented in a longitudinal direction, with circumferential myofibrils present only in the thinner subepicardium [13]. Consequently RV contraction is effectively longitudinal with ejection propagating from apex towards outflow tract, and rotational deformation being only a minor component of RV contraction [14]. During RV contraction, ventricular length shortens and the base is translocated towards the apex. Analysis of this dynamic ventricular movement is used to assess both contractile function and diastolic relaxation. During systole the tricuspid annular plane descends towards the apex, which itself remains relatively stationary [15]. Tricuspid annular plane systolic excursion (TAPSE) provides an approximation of RV longitudinal contractility, and can be measured using M-mode or with pulse-wave tissue Doppler imaging (S, E’ and A’ waveforms). With this approach Linqvist at al reported RVOT fractional shortening (RVOTFS) to correlate with both RV longitudinal function as measured by TAPSE, and pulmonary arterial pressure [16]. TAPSE, however, was not performed in this study since this it is appropriately measured using the more expanded apical 4-chamber view.

2D-strain is a further analysis of cardiac motion based on the tracking of “speckle” movement within the myocardial wall during the cardiac cycle. Naturally occurring bright speckles within the myocardium act as acoustic markers for tracking, and are generated by backscatter signal during ultrasound examination [15]. Analysis is offline and based on commercial software algorithms. Strain (ε) is calculated from the relation L—L_o_ / L_o,_ where L is the final length and L_o_ the initial length of a myocardial wall speckle. Strain measures vector movement in relation to myofibril architecture: deformation is negative with longitudinal and circumferential strain, and positive for radial strain relating to wall thickening. Strain rate is the velocity of deformation, or ε/time between frames.

The value of LV global longitudinal strain (GLS) is evident in clinical studies, where strain deformation has an earlier prognostic value than ejection fraction, particularly in predicting major adverse cardiac events [17]. Strain analysis of the RV is similarly reported to identify early changes in RV function before maladaptive remodelling becomes evident. In patients with pulmonary hypertension for example, longitudinal strain is segmentally decreased despite normal global RV function [18]. With respect to animal models the application of 2D-strain is emerging, particularly in the assessment of left ventricular function [6, 19, 20]. However, the application of RV strain to animal models is not previously reported, but as with clinical studies, has major potential in characterizing RV function. This study has demonstrated 2D-strain analysis of the rodent RV is feasible. However, we are aware that limitations do exist. Firstly, strain analysis was suboptimal in many animals due to poor endocardial border definition and may be specific to our current ultrasound technology. Secondly, the MRV represents a “hybrid SAX” view with measurement of circumferential, radial (or longitudinal) wall deformation components being dependant on RV myofibril orientation, relative to the ultrasound beam. Further investigation of strain analysis using the MRV view is required to define normal values in rodents.

The use of animal models of disease has direct translational value in assessing the efficacy of potential therapeutic agents [3]. In COPD, for example, it is recognized that 40% of deaths in COPD result directly from associated cardiovascular disease and particularly from right heart (RH) dysfunction [21–24]. In many patients, mild to moderate pulmonary hypertension (PH) is common [25], with mean pulmonary arterial pressure (mPAP) between 25 and 30 mmHg [26]. The adaptive response of the right ventricle to increased pulmonary pressure includes chamber dilatation, wall hypertrophy and systolic dysfunction [27]. The presence of PH in COPD is associated with a poor prognosis and reduces survival [28]. In this respect assessment of right ventricular function using 2D-echocardiography remains an important aspect of clinical management [29].

A limitation to this study was the variation in image quality between animals. Technical accuracy is dependent on anatomical recognition and orientation, which ensures standardization of echocardiographic views. Incomplete or oblique views, poor definition of endocardial or epicardial borders, as well as Doppler mal-alignment, are all potential sources of error. Our experience indicates incomplete imaging to occur in only 1 of the 15 animals. However strain analysis was particularly affected by poor border definition and high frame rates, and prevented analysis in many animals. With respect to gel injection and RV wall dissection at autopsy, a potential error was the failure to index measurements to animal body surface area. A further limitation in methodology is that CO was not validated with ultrasound measurement of PA flow. However our values were consistent with reported values. Transthoracic examination was well tolerated, with hemodynamic stability throughout anesthesia.

In summary, we have demonstrated that examination using the MRV and RVOT views has the potential to provide a comprehensive assessment of the RV particularly when standard 4C or 2C apical views are often suboptimal. These views enable a large section of the RV free wall to be imaged, enabling assessment of dilatation, wall hypertrophy and strain analysis. Further examination of the RVOT allows RV morphology, and contractile function to be serially assessed. In rodents 2D-strain imaging of the RV is feasible, and with appropriate study design has the potential to identify early systolic dysfunction.

## Supporting information

S1 Text Echocardiography spreadsheet containing raw data. (XLSX)
